# Enhancing Knowledge Flow in a Health Care Context: A Mobile Computing Approach

**DOI:** 10.2196/mhealth.2543

**Published:** 2014-11-26

**Authors:** Jonice Oliveira, Diego Da Silva Souza, Patrícia Zudio de Lima, Pedro C da Silveira, Jano Moreira de Souza

**Affiliations:** ^1^Graduate School in Computing Science (PPGI)Federal University of Rio de Janeiro/Universidade Federal do Rio de Janeiro (UFRJ)Rio de JaneiroBrazil; ^2^Department of Computing Science (DCC), Institute of MathematicsUniversidade Federal do Rio de Janeiro (UFRJ)Rio de JaneiroBrazil; ^3^Systems and Computer Engineering Graduate School (COPPE)Universidade Federal do Rio de Janeiro (UFRJ)Rio de JaneiroBrazil

**Keywords:** knowledge sharing, health care, mobile computing, Medicine 2.0, collaborative interaction, social computing

## Abstract

**Background:**

Advances in mobile computing and wireless communication have allowed people to interact and exchange knowledge almost anywhere. These technologies support Medicine 2.0, where the health knowledge flows among all involved people (eg, patients, caregivers, doctors, and patients’ relatives).

**Objective:**

Our paper proposes a knowledge-sharing environment that takes advantage of mobile computing and contextual information to support knowledge sharing among participants within a health care community (ie, from patients to health professionals). This software environment enables knowledge exchange using peer-to-peer (P2P) mobile networks based on users’ profiles, and it facilitates face-to-face interactions among people with similar health interests, needs, or goals.

**Methods:**

First, we reviewed and analyzed relevant scientific articles and software apps to determine the current state of knowledge flow within health care. Although no proposal was capable of addressing every aspect in the Medicine 2.0 paradigm, a list of requirements was compiled. Using this requirement list and our previous works, a knowledge-sharing environment was created integrating Mobile Exchange of Knowledge (MEK) and the Easy to Deploy Indoor Positioning System (EDIPS), and a twofold qualitative evaluation was performed. Second, we analyzed the efficiency and reliability of the knowledge that the integrated MEK-EDIPS tool provided to users according to their interest topics, and then performed a proof of concept with health professionals to determine the feasibility and usefulness of using this solution in a real-world scenario.

**Results:**

. Using MEK, we reached 100% precision and 80% recall in the exchange of files within the peer-to-peer network. The mechanism that facilitated face-to-face interactions was evaluated by the difference between the location indicated by the EDIPS tool and the actual location of the people involved in the knowledge exchange. The average distance error was <6.28 m for an indoor environment. The usability and usefulness of this tool was assessed by questioning a sample of 18 health professionals: 94% (17/18) agreed the integrated MEK-EDIPS tool provides greater interaction among all the participants (eg, patients, caregivers, doctors, and patients’ relatives), most considered it extremely important in the health scenario, 72% (13/18) believed it could increase the knowledge flow in a health environment, and 67% (12/18) recommend it or would like to recommend its use.

**Conclusions:**

The integrated MEK-EDIPS tool can provide more services than any other software tool analyzed in this paper. The proposed integrated MEK-EDIPS tool seems to be the best alternative for supporting health knowledge flow within the Medicine 2.0 paradigm.

## Introduction

### Background

The way we communicate with the world is changing every day because of advances in wireless technologies. Mobile devices such as cell phones, netbooks, and tablets allow us to establish permanent connection and interaction with other people, almost anywhere. This communication allows people to constantly exchange knowledge among themselves. Indeed, most daily human activities have been transferred from personal computers to mobile devices [1]. People chat on their phones wherever they are, share snapshots of interesting places taken with smartphones, and regularly send and receive text messages.

The dissemination of wireless devices and their increasing use have created a huge network that has changed the way people communicate. These interactions seem to have no limits with regard to space and time. An Internet connection is often the only requisite for people to be able to work, interact, or entertain themselves, anywhere and at any time [1]. Nowadays, the interactions among people are easier, faster, and more frequent than they were just a few years ago. The interval between messages in asynchronous communication has become inconspicuous to regular users. Moreover, people are now more open to interact with other people they do not know to help them address a specific problem. These spontaneous links are weak and they are usually lost at the end of the activity [1].

### Medicine 2.0

The new interaction scenario can be considered to be a part of what has been called *cyberculture* [2]. This scenario encompasses people and objects in an immense and interconnected environment that is changing the way people interact with the spaces; thus, the world takes on new dimensions [3]. This new interaction paradigm has also reached the health care area, where information technology is changing medical practice and research, and empowering those who need quick access to supporting health information. It includes a long list of participants who play several roles in health care processes. This networked scenario has been called *Medicine 2.0* [4], and it can be understood as:

Web-based services for health care consumers, caregivers, patients, health professionals, and biomedical researchers that use Web 2.0 technologies, semantic Web, or virtual-reality tools to enable and facilitate specifically social networking, participation, apomediation, collaboration, and openness within and between these user groups. [5]

**Figure 1:**
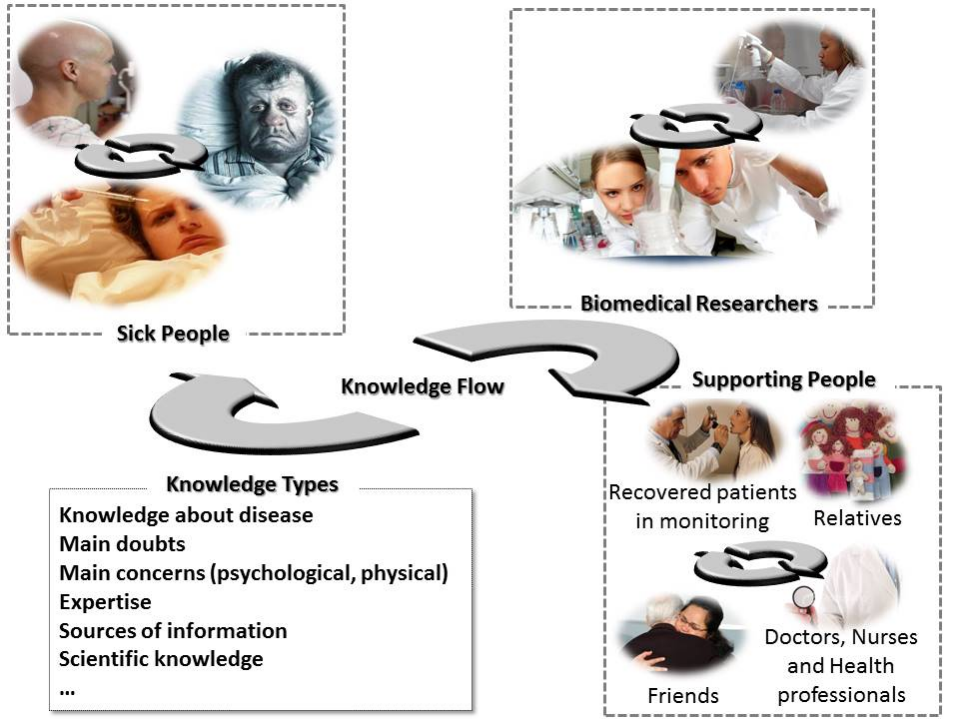
Knowledge flow in a medical scenario that adheres to Medicine 2.0.

There is a powerful knowledge flow in the health care scenario (see Figure 1) and the Medicine 2.0 principles, services, and applications are used to support it. When knowledge flow and these interactions are lacking—particularly toward patients and their families—it may have a negative effect on patients and prolong recovery time [6]. Medicine 2.0 tries to mitigate this situation by generating a social environment where people are more interconnected and available to support one another.

In Figure 1, we can identify 3 groups of participants: sick people/patients, supporting people, and biomedical researchers. These participants create different pieces of knowledge and develop different degrees of expertise that can eventually be shared with others in need of external support.

The patients are those undergoing treatment who are eventually able to provide information about their own illness, symptoms, physical and psychological reactions, and previous diseases and treatments [7]. During treatment, several subgroups are involved (health staff such as doctors, nurses, and other health professionals) who are responsible for diagnosing and treating patients. They have solid and reliable domain knowledge that can be shared to support not only patients, but also their families and caregivers. Such knowledge is useful to clarify doubts and concerns about a disease and its treatments, and instructing the supporting people on how to deal with the patients.

We also have patients who have already recovered, but are still being monitored. This is typical when people are in long-term care (eg, cancer treatment). These patients are a special kind of knowledge provider because they can share information on the disease and its treatment in an easy, informal, and understandable way. This is particularly important for other patients and their families and friends, all of whom usually appreciate the support of people who have been in a similar situation.

Understanding this knowledge-sharing scenario can help software engineers to create new types of solutions. If these pieces of knowledge can freely flow among all members of this huge network, we can create collective knowledge that can and should be harnessed to streamline the treatments and care procedures, improving the quality of life for patients.

As shown in the model defined by Eysenbach [4], 5 major concepts that emerge from Web 2.0 were applied to health care: social networking, participation, apomediation, collaboration, and openness. These emerging and recurring concepts, which are the basis of Medicine 2.0 [4], will outlive the specific tools and services that should be offered to the end users (ie, people participating in health care processes).

The term “social network” has become very popular with the growth of tools intended to manage relationships or disseminate news (eg, Facebook, Google+, and Twitter). These tools, which are based on the principles of Web 2.0, can be used in a medical scenario to facilitate interactions among participants, thus helping prevent diseases through the dissemination of health information and motivating users to take responsibility for their own health situation.

However, the real meaning of social networking is the interactions among a set of actors who may have different kinds of relationships with one another. A social network may have a few or many members (ie, nodes) and 1 or more types of relationships (ie, edges) between the members [8]. By analyzing and understanding how and when people interact with one another, we can determine the propagation of diseases [9-14], how science is developed [15], and how it could be improved through appropriate alliances [16]. The analysis of social networks can help disseminate information, determine the quality of a piece of information, and enable collaborative filtering. People are usually less reluctant to accept a piece of information when it is offered by a community member as opposed to an unknown person. Typically, it is assumed that relevant people are linked to reliable information as well. These links among people can be used to turn an information campaign into a success (eg, to alert about a new treatment, problems with a medication, an epidemic season, or regular exams).

Figure 1 shows how knowledge flows through interactions among the members of a particular health care community. The links among participants allow each piece of knowledge to be enhanced, updated, or transformed during each interaction.

In Medicine 2.0, the main concept directly related to social networking is participation. Members of a community are free to participate, connect, and cooperate with one another, which is reflected by the various levels of people’s participation. Consequently, this generates unique and unprecedented opportunities for engaging patients in their own health care activities, in the construction of knowledge regarding a treatment, and in connecting people using informal and formal knowledge.

This complex network leads to another concept of Medicine 2.0. Apomediation represents the access to knowledge without intermediaries such as health professionals giving “relevant” information to a patient [4]. Eysenbach [4] stated:

In the age of Web 2.0, there is a special form of disintermediation: an information-seeking strategy in which people rely less on traditional experts and authorities such as gatekeepers, but instead receive guidance from apomediaries; that is, networked collaborative filtering processes. The difference between an intermediary and an apomediary is that an intermediary stands between the consumer and the information, focusing on the need for a mediating agent to receive, validate, and pass on the information. By contrast, apomediation means that there are agents (eg, people or tools) who stand by to guide a consumer to high quality information and services without being a prerequisite for obtaining that information or service. [17,18]

Collaboration is the fourth major concept linked to Medicine 2.0. It represents the actions that allow the connection of communities that follow similar goals or have similar interests. Finally, the openness concept is related to the interoperability (for data and services) among the software systems used by the participants in health care activities according to the interaction paradigm proposed in Medicine 2.0.

We believe that mobile computing is an important driver for Medicine 2.0 because of the widespread use of mobile devices and their ability to capture contextual information. By contextual information, we mean the meta-information that can be used to trigger knowledge exchange between 2 people (eg, information that specifies the location or the proximity of people who are looking for knowledge that another person can provide them). Based on positioning information (ie, contextual information), mobile devices can provide relevant data about the place where users are located, the events that are occurring there, and other community members who are nearby at that time.

According to the Medicine 2.0 paradigm, the use of mobile devices and contextual information can enhance the interactions and knowledge exchange among members of a health care community. To validate this hypothesis, we developed and evaluated a software environment that uses mobile devices (particularly smartphones) and contextual information for such a purpose. In this paper, we identify the current status in apps that support Medicine 2.0 and describe and validate the proposed system.

## Methods

### Creation of the Computational Solution

**Figure 2:**
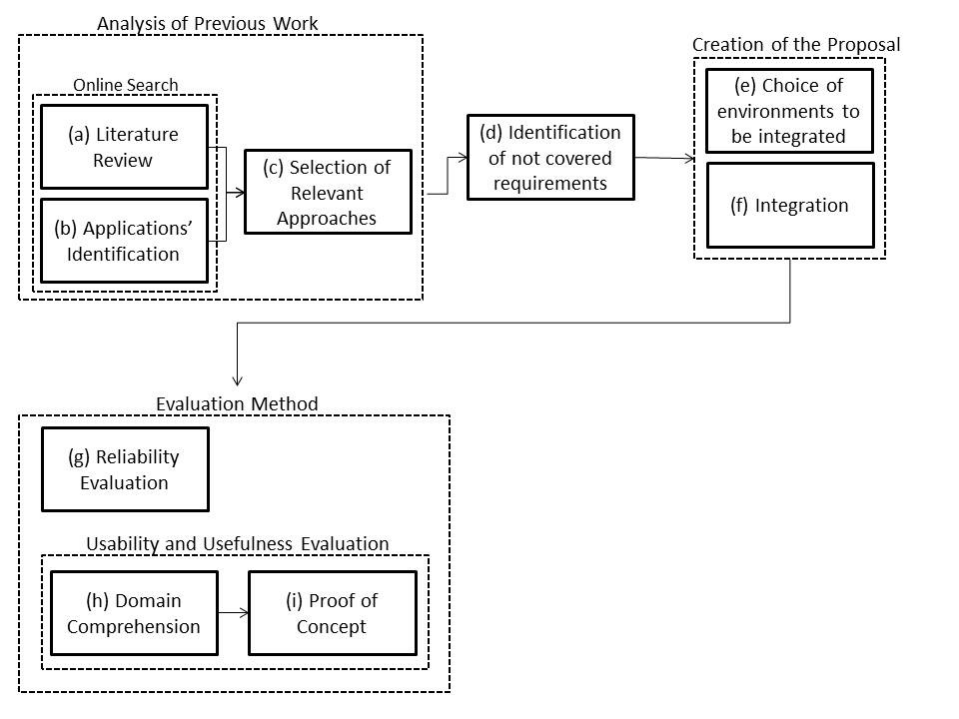
The creation process of the knowledge flow solution.

The conception of the knowledge-sharing environment involved an evolving process that included several steps (Figure 2). First, we analyzed previous works to determine the current state of knowledge sharing as a support to the health care process. In particular, we reviewed the scientific literature and analyzed the main software apps that could be used to address the problem of knowledge sharing in a medical scenario. Then we identified a set of scientific proposals and apps, considering also the main themes addressed by Medicine 2.0. Based on this analysis, we identified the requirements of the health care scenario that have not been addressed by the reviewed proposals.

An initial knowledge-sharing environment for mobile devices was developed based on the previous works and the requirements of Medicine 2.0 that were not addressed by the proposals presented in the literature or on the market . The development process involved 2 steps: the selection of already implemented and complementary products and the integration of the selected tools.

The resulting knowledge-sharing platform underwent a twofold evaluation. First, we analyzed the efficiency and reliability of the answers (ie, knowledge provision) that the system gave to people asking questions about illnesses or treatments and the identification of experts’ locations. Second, we evaluated the suitability of the environment to support knowledge acquisition and exchange in a particular domain. We also performed a proof of concept to determine the feasibility of applying this solution to a real-world scenario.

### Analysis of Previous Works: Literature and Apps

#### Online Search

A search to identify works related to knowledge exchange in health care scenarios with themes from Medicine 2.0 and eHealth was conducted in January 2013. Initially, we only considered scientific works published in proceedings, book chapters, and journal articles over the previous 3 years (Figure 2). The keywords used in this search were mHealth, health care, eHealth, mobile, collaboration, social network, apomediation, participation, knowledge dissemination, and Android. Other nonmedical sources were used in subsequent searches; in particular, publications from the Institute of Electrical and Electronics Engineers (IEEE) Computer Society Library were also considered. The keywords used for these subsequent searches were mHealth, health care, eHealth, medical service, knowledge dissemination, and opportunistic collaboration.

After collecting the relevant publications, we extended the search to include mobile apps that could be used to support some of the main concepts of Medicine 2.0 (Figure 2). We performed a search within Apple iTunes [19] and the Google Play Store [20] using the following terms: eHealth, health care collaboration, medical collaboration, medical knowledge dissemination, health care knowledge dissemination, and opportunistic collaboration. In both software repositories, we searched primarily for freeware apps because they could eventually be integrated into other solutions (without cost to the end users) to build a knowledge-sharing environment. We also looked for tools and projects related to knowledge exchange using mobile devices that were not specifically designed for health care, but that could be used to support Medicine 2.0.

We also searched for services by using the keywords “Web services” and “health.” This search gave us primarily social networks designed to support patients or health professionals.

#### Selection of Relevant Approaches

##### Overview

The articles, apps, and programs obtained in the previous step were analyzed from the standpoint of the 5 major themes of Medicine 2.0 (ie, social networking, participation, apomediation, collaboration, and openness).. Those that did not support at least one of the key Medicine 2.0 themes were excluded. For the apps, only freeware tools were chosen for a more detailed analysis. Applying these selection criteria, we built a study corpus composed of 32 relevant articles (describing solutions and projects) and 133 mobile apps. This is step c Figure 2.

These were carefully reviewed to determine whether the following themes were supported or not. The themes were defined based on both the recommendations for supporting Medicine 2.0 apps and our experience in developing these types of apps.

##### Social Networking

By using the identification of relationships and interaction types, we can characterize the relationships among members as, for example, 2 members who are family members, coworkers, neighbors, patients, caregivers, or patients undergoing treatment. More than 1 relationship type can characterize the link between 2 people. Distinguishing the types of relationships between community members and the frequency of their interactions is required to understand what is going on in a community.

Analysis of the network structure identifies the roles that are present in a social network, the number of members per role, and the interaction links. On the basis of these findings, the size and structure of the community can be established.

Analysis of an egocentric network recognizes people linked to a particular member, the roles of these people, the relationship types, and the frequency of interactions with the observed member.

In social network analysis, several graph metrics are used (eg, centrality, density, and distance between members). Using predefined graph metrics, we analyzed the proposals to determine if they provide functionalities to calculate these graph metrics.

Identification of relevant members allows relevant members of a community to be identified (eg, leaders or experts). Usually, the life of these networks depends on these people. Counting on an important number of relevant people helps keep the community alive. The relevance is a qualification given by the community members based on the attitude and value of the contributions of each person.

Identification of content and main topics analyzes the content that triggers the members’ interactions and, based on the findings, the main subjects discussed in the community are determined.

##### Participation

Asynchronous communication allows 2 or more community members to interact sending messages or data intermittently rather than in a steady stream. For example, emails, SMS or file transferring, where users do not need to be connected in the same time to communicate to each other. Differently, we have Synchronous communication (synchronous communication), as in chats or telephone/voip calls, where all the users need to connect to communicate.

Using the service mechanisms for encouraging participation, we can implement incentive mechanisms focused on encouraging participation inside a community. There are several approaches for implementing these incentives, such as social incentives (eg, status and power) and intangible rewards (eg, esthetic improvement or public recognition). Depending on the current needs of the community, adaptive rewards can also be implemented.

Participation also involves the service promotion of various kinds of participation. Some communities support particular activities that a member (or group of members) offers to other community members (eg, physical or virtual resources, online or face-to-face meetings, or specialized talks). Counting on services that support these kinds of (special) participations helps keep the community alive and helps it to evolve based on its own interests.

Security and privacy of the user’s personal information protects community members from unauthorized use of their personal information. It also considers information about the users’ activities in the network.

Information protection allows users to define levels of visibility for their information (eg, public or private information, information accessible only to friends or family).

Interest identification allows identification of the interest areas of a community member. Thus, a software application can facilitate knowledge exchange among people with similar interests.

Expertise identification identifies the expertise area of each community member. This information can then be used by a supporting application to suggest potential experts on a specific topic.

Attention level provides status awareness, indicating the current availability of a certain community member (eg, available, busy, or disconnected). Typically, this awareness mechanism can support synchronous and asynchronous interactions among these people.

##### Apomediation

Autonomous operation is a self-management service that provides supporting information to people who require it. The information delivered by this service is based on the pieces of knowledge generated by the community.

Apomediation considers that knowledge is neither centralized nor held by intermediaries, such as experts or authorities. By using decentralized environment, we evaluated the presence of services that allow people to produce, keep, and consume the shared knowledge in a decentralized way.

Informal learning supports people learning through active participation in the community. Typically, this service supports people interaction and information exchange. Sometimes this learning process involves unofficial information sources.

Source credibility indicates the credibility level of a person based on the opinion of other community members. People with high credibility are asked questions more frequently by other community members in need of external support.

Message credibility indicates how much support (credibility) a certain message has. The credibility of the community member who delivered the message tends to be more relevant for other community members compared to the formal citations.

Information filtering allows relevant information to be selected based on a filtering process that usually considers several criteria. This filtering process can be carried out using several strategies (eg, applying information retrieval techniques or through the collaborative participation of community members).

Detection of opinion leaders identifies people who are leaders of opinion. Typically, these people polarize and segment the community opinions; therefore, their interventions should sometimes be mediated.

##### Collaboration

Location awareness allows computing devices to determine their location in indoor and outdoor environments. This is particularly useful to promote face-to-face meetings between people (eg, in a hospital).

Contextual information implements other awareness mechanisms that provide relevant information to understand, to perceive, to feel or to be conscious of events, objects or sensory patterns (in addition to the user’s location) such as user presence and availability, or similarity of users’ profiles.

Opportunistic collaboration support provides communication and interaction support for users who have decided to start a spontaneous computer-mediated interaction (eg, based on topics of common interest).

##### Openness

Various content formats allow an application to use different data and media formats, ensuring data interoperability among supporting services or applications.

Semantic integration allows a software system to perform data integration using semantic mechanisms, facilitating knowledge searches and inference processes.

Transparency represents the ability to access data easily, regardless of its original sources and the application that created it.

Free access indicates that no charges will be made for accessing the shared knowledge or for getting external support.

By using this analysis, we could identify functionalities that were not covered by current approaches of knowledge sharing in medical scenario. These services were used to build the comparative study between the proposals in the literature and apps review, and to guide the development of a new supporting system, described subsequently. The results of this comparison are detailed in the section Results.

### Creation of the Proposal

Aiming to provide complete support for knowledge flow in medical scenarios (as shown in Figure 1) and to use contextual information to enrich the access and creation of knowledge, we designed a new knowledge-sharing supporting system. The first intention was neither to create a completely new solution, nor to use closely tied tools that could make future extension of the system difficult. Instead, we opted for the integration of already implemented solutions. To determine which software to integrate, we defined a set of requirements the candidate systems had to satisfy:

1. The source code is available (open or granted) to enable future customizations.
2. Complete documentation available and development teams willing to collaborate by providing technical support when needed.
3. The existence of projects with scientific and academic background, where the new customizations related on knowledge sharing and advances made on them could be continued by future research efforts.
4. A product that is already being used in the health domain.
5. Possibility of integration with other products.
6. The integrated products should address most themes of Medicine 2.0.

With this criteria, we based our proposal on the integration of mobile exchange of knowledge (MEK) [21,22] and the Easy to Deploy Indoor Positioning System (EDIPS) [23].

### Evaluation of the Integrated Knowledge-Sharing Solution

#### Goal

The goal of the evaluation was twofold: to assess the correctness of the answers provided by the integrated MEK-EDIPS tool (ie, evaluate the system’s reliability) and to identify the intended use (ie, usability and usefulness evaluation).

#### Reliability Evaluation

**Figure 3:**
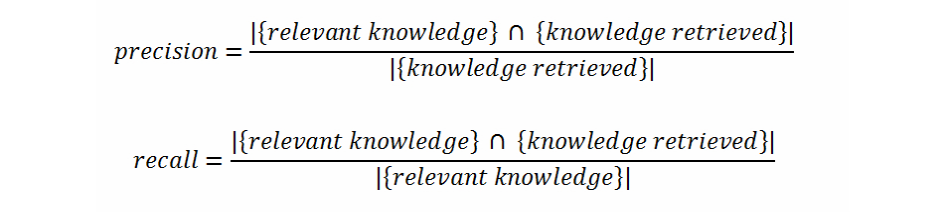
Precision and recall metrics.

In this stage, we analyzed MEK and EDIPS separately. For MEK, which is a peer-to-peer (P2P) platform for disseminating information, we simulated some scenarios for information sharing using 6 smartphones. These phones had similar configurations and features. Based on the knowledge exchanges performed in the simulated scenarios, we calculated the precision and recall metrics (Figure 3).

The simulated scenarios represented different ways of sharing knowledge, starting with a simple exchange that involved only 2 peers. The network was gradually increased one by one until a network with 6 mutually interacting nodes was achieved. In this evaluation phase, we used 30 pieces of knowledge from various areas of interest.

In MEK, a piece of knowledge is any resource that can be exchanged, such as a piece of text, or an archive with its description. Two evaluations were performed for each scenario, involving different people. The first scenario involved the exchange of knowledge pieces without attached files. In the second scenario, the pieces of knowledge had attached files ranging from 1 to 5 MB. The analyses of the knowledge dissemination were performed based on the following metrics:

1. Number of failed connections. This metric indicated the number of interaction attempts for which no matching peers were found.
2. Number of connections without transfer. This quantifies the number of times that a device found a peer, but no knowledge exchange occurred.
3. Number of successful transfers. This metric is similar to the previous one; however, it counts the number of times that knowledge was exchanged between peers.

For the EDIPS, a mobile application that identifies the presence and location of people in indoor environments, the evaluation process was quite different. The EDIPS evaluation was conducted on the third floor of the Computer Science Department of the University of Chile. It has an area of 1320 m^2^ (55 m × 24 m) with 6 Wi-Fi access points. The devices used in this evaluation were smartphones and all possessed HTC Diamond Touch 2.

**Figure 4:**
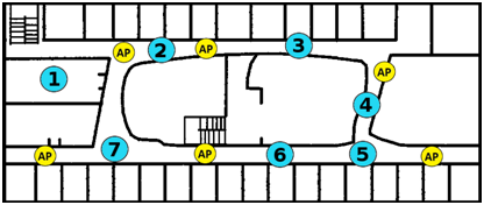
Map of the testing area with Wi-Fi access points (AP) and the estimated measuring locations (1-7) [19].

This evaluation analyzed the efficiency of the user location prediction made by the system. The location prediction error was the distance (in meters) between the user’s actual location and the position estimated by EDIPS. Figure 4 shows the map of the physical infrastructure where the evaluation was performed. We calculated an average location error by using 25-35 samples at each point.

#### Usability and Usefulness Evaluation

##### Phases

This suitability evaluation was done in 2 phases. During the first phase,where we analyze the domain, we identified knowledge acquisition as a main limitation in a health scenario. In this phase, we also collected data to understand how health professionals acquire knowledge, what they consider important information during treatment, and how their patients learn about a disease and its treatment. In the second phase, we tried a proof of concept of the MEK-EDIPS tool, where we presented the system to health professionals and they assessed it.

##### Domain Comprehension

To evaluate the domain comprehension, a goal, question, metric (GQM) [24] template was used. Based on this template, we analyzed the knowledge acquisition process in a health scenario, especially during a treatment, for the purpose of evaluating the efficiency of the knowledge acquisition process, and the reliability of the sources and the services used by professionals and patients with respect to (1) main information sources and usage frequency, (2) importance level of information in a treatment, (3) effort required to acquire information from the patient, and (4) usability and reliability level of the information consumed by patients from the viewpoint of health professionals.

All the participants were volunteers. First, we explained the study and all its stages and some health professionals agreed to participate in it. The sample was analyzed by gender, age, profession, expertise area, institution where the person works, main work area (clinical, research, management, or other), experience (calculated in years since graduation), time (in years) that a participant had been working at the current institution, and education level.

No training was needed for this study. All participants received the link to an online questionnaire that included questions in different areas.

The questions about sources of knowledge acquisition identified the main resources used to acquire new knowledge and the frequency the health care workers accessed them. The types of resources were classroom/face-to-face courses, distance courses, lectures, textbooks, scientific articles, discussion with more experienced professionals, discussion with less experienced professionals, discussion with professionals outside of my area, presentations in scientific meetings, empirical observation of other professionals, specialized websites, study groups (face-to-face or virtual), social networks or media (eg, Facebook, Twitter, LinkedIn), and others (specified by the participant). The frequency of access to these information sources were at least once a year, 2-6 times a year, 1-3 times a month, 1-3 times a week, or daily.

The questions about importance of information in a treatment identified the significance of information provided by patients during a treatment and were rated as not important, somewhat important, important, very important, or extremely important. The types of information considered in the questionnaire were symptoms, doubts about the disease, fears about medication or treatment stages, physical reactions, psychological reactions, previous diseases, treatments already undertaken, routines, hobbies and information about private life, religious beliefs and superstitions, details about work (eg, location, infrastructure, and level of violence), details about residence (eg, location, infrastructure, basic sanitation, transportation, and level of violence), educational and cultural background, and others.

The questions about ease in acquiring information from patients asked how easy it was to get prior information from patients. The answers were difficult, somewhat easy, easy, very easy, or extremely easy.

The questions about usability and reliability of the information consumed by patients identified the most common information sources used by patients and how the health care workers evaluated the reliability of these sources. The usage level was rated as very little, little, regular, frequent, or very intensive. The source’s reliability was rated by health care workers as unreliable, not very reliable, reliable, very reliable, or extremely reliable. The types of information source used by patients were divided into scientific publications, other health professionals, friends and relatives, known people who have had the disease, friends and relatives of people who have had the disease, social networks or media, specialized virtual communities, webpages and other Internet materials, and others.

The following specific question was posed to the participants about general feelings about knowledge exchange: Do you believe that greater interaction among patients, health professionals, researchers, recovered patients, and caregivers brings benefits to a patient’s treatment and quality of life? For this question, participants could answer yes or no.

In this phase, we only wanted to understand the knowledge exchange scenario; we did not control or measure the quality of the knowledge exchanges or determine the efficiency and effectiveness of this process.

##### Proof of Concept

After collecting information to understand how professionals acquire knowledge, what they consider important information during a treatment, and how their patients learn about their disease and its treatment, we did a proof of concept. In this qualitative study, conducted in January 2013, we presented the knowledge-sharing environment to health professionals for evaluation. As defined in GQM [24], this study was to analyze the functionalities of the system for the purpose of evaluating the support provided to the knowledge flow in a health scenario with respect to possible uses of the environment by different actors in a hospital, its applicability in a health scenario, and the possibility of recommendation and use, from the viewpoint of health professionals, in the context of the MEK-EDIPS proposal.

The participants were the same as in the knowledge acquisition evaluation. They received brief training, where we described the MEK-EDIPS environment and its main services. Then participants answered a questionnaire about the knowledge-sharing program (Textbox 1).

Although the sample was characterized by different attributes, the most relevant ones were the experience level, particularly the time (in years) since graduation and the time (in years) that working at the current institution, education level, and the main area of work (eg, clinical, research, management, or others). The attributes that delineate this experiment (ie, the dependent variables) are possible use (questions 1, 2, and 5) and increase of knowledge flow (question 4).

We hypothesize that there are recognized possibilities for using the integrated MEK-EDIPS tool (ie, its use is considered important and would be recommended) and that the integrated MEK-EDIPS tool can increase knowledge flow in a health scenario.

In this study, the hypotheses about possible use and knowledge flow are equally important. In the same way, its usage is only relevant if there really is better knowledge flow among actors.

**The questionnaire used to analyze the benefits of MEK-EDIPS environment in knowledge sharing**

(Q1) Possible uses of the environment by different actors in a health care process: For each possible use, the interviewees rated the importance level as a little important, somewhat important, important, very important, or extremely important. The potential end users of the system are as follows:

- Patients and relatives: Obtain information on the disease for additional understanding and, consequently, better treatment.
- Patients and relatives: Comfort of knowing, meeting, and collaborating with people who are going through (or that have gone through) the same illness.
- Patients and relatives: One way to get information that is more reliable than that obtained from the sources they normally use.
- Health professionals: Possibility to expand their knowledge more easily through access to scientific articles, experimental results, and treatments shared by other colleagues.
- Health professionals: Obtaining information that may help in the treatment that is usually omitted in consultations (eg, major doubts, unreliable data the patient may be relying on, reactions, or beliefs).
- Researchers: Collecting information or results to help them create new hypotheses for further research.
- Managers: Improve the provision of health information. The knowledge-sharing environment can identify the areas most people are interested in. The system can also support knowledge exchange and detect areas where there are information gaps.

(Q2) Other usage possibilities: The interviewees could indicate any other kind of usage different from that previously mentioned.

(Q3) Advantages and disadvantages of using the platform: The interviewees could indicate the environment’s strengths and weaknesses.

(Q4) Impact of the system: The participants were asked to answer the following question: Do you believe that this environment can increase the flow of knowledge in the medical scenario? The participants answered using a 5-point scale, where 1=no and 5=absolutely.

(Q5) Users’ acceptance of the system: The participants were asked to answer the following question: Would you use the platform or recommend its use? The participants answered using a 5-point scale, where 1=never and 5=absolutely.

(Q6) Free space for any comments: This space allowed the participants to indicate any other information that they considered relevant about the usage of the proposed environment.

### MEK-EDIS: The Integrated Environment to Aid Knowledge Exchange

#### Mobile Exchange of Knowledge

**Figure 5:**
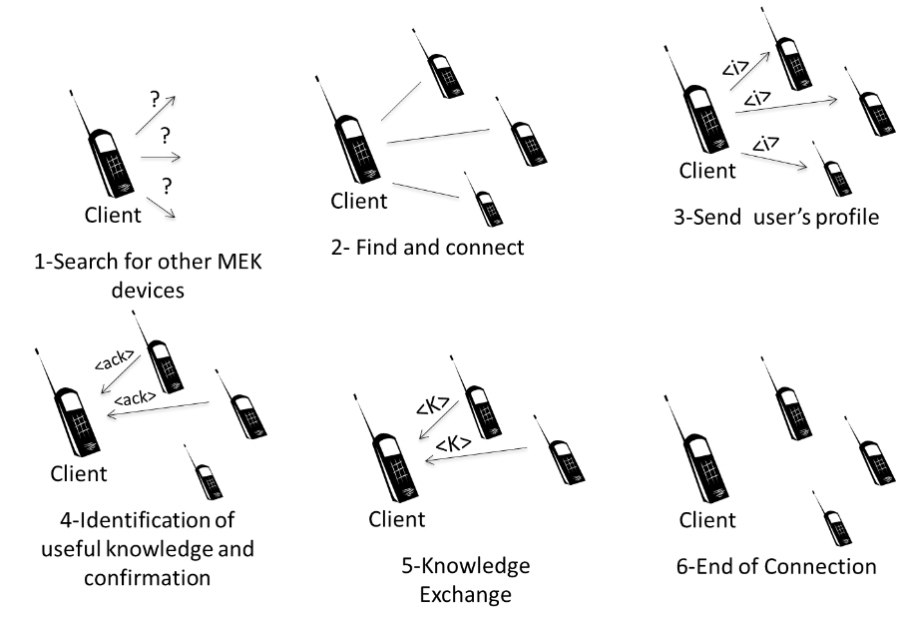
How knowledge is exchanged in Mobile Exchange of Knowledge (MEK) [22], where is information about user’s profile, <ack> is acknowledge and <k>is a piece of knowledge.

The main purpose of MEK is to disseminate knowledge in a proactive and viral way. In this paper, we consider knowledge to be any information, such as images, texts, or audio, that can be scanned or created digitally. Figure 5 shows how the environment allows the interconnection of devices and the exchange of pieces of knowledge between them. This communication process uses Bluetooth. This design decision was made because this network protocol is present in most mobile devices and its usage is widespread. Moreover, no communication infrastructure is required to use it.

Our motivation for developing this proposal came from the hypothesis that people who have easy access to information become a great source of knowledge, thus avoiding the constraint of having just one person or group as an information source. Therefore, MEK aims to increase the exchange of knowledge among people, forming a network composed of users sharing the same interests.

After installing MEK on a smartphone, users have to identify their interests by filling in a small form, indicating their areas of interest, relevant keywords, and other information that will be used to build their personal profile. This profile will be then used to support knowledge exchange processes. The interest areas are based on a preset taxonomy, structured as a tree. After building the user profile, users could subscribe to knowledge topics delivered to their mobile phone. This knowledge will be rated according to the previously mentioned tree and the keywords from the user in the system to help improve knowledge classification.

For knowledge exchange, the MEK system of a device periodically scans for other mobile phones in the vicinity that are running the application. When another device is found, the profiles of the local users, their areas of interest, and keywords are exchanged. If there are any matches, the selected knowledge is sent to the requester (see Figure 5).

#### Easy to Deploy Indoor Positioning System

The EDIPS [23] is a mobile application that maps the position of several people in a closed environment. This system uses a mix of inertial navigation and wave analysis to perform the positioning process..

##### Inertial Navigation Systems

The inertial navigation positioning method uses inertial sensors (eg, gyroscopes and accelerometers) to capture the movement of a user in indoor environments. A positioning system that is based solely on these sensors becomes imprecise over time because the sensors carry a margin of error that accumulates whenever a new measurement is made. Nevertheless, it is an excellent support tool that can be combined with other techniques to achieve simplicity and accuracy.

##### Wave Analysis

The wave analysis positioning method is based on the analysis of a network’s signal strength. It uses multiple reference points that emit signals to a receptor device, which then (based on the wave properties) infers its position in the physical environment. The 3 common techniques for wave analysis are proximity detection, signal triangulation, and fingerprinting.

Proximity detection uses device detectors that are placed on previous known positions. When a resource is identified by one of these detectors, its coordinates are reported to a component in charge of mapping the device. Although this strategy could be very accurate for detecting objects in motion, it requires the use of specialized hardware and significant effort to prepare the environment where the positioning system would be used.

Signal triangulation uses the geometric properties of the triangle to estimate the position of a resource in a 2-dimensional scenario. The estimation process uses 3 reference points with a known location from which signals are emitted. The signals are then surveyed by the device whose location is to be estimated. By estimating the distance between the device and each reference point and triangulating the signals, it is possible to pinpoint the resource in the area within a fairly reasonable margin of error. The advantage of this method is that it usually requires little setup effort to be used in an indoor environment.

Fingerprinting estimates the position of a resource in the environment, comparing the signal strengths detected by the receptor device against a set of prestored signals that correspond to different points in the physical area. This strategy usually has 2 phases: the online and the offline phase. In the online phase, several signal samples are collected from multiple reference points at the physical locations. This information is used to make a grid, where each cell is characterized by the set of signal strengths that can be detected at the location. In the online phase, the signal strengths captured by a device are then compared to the information preset in the offline phase. Comparing both sets of signals, the method estimates the current position of a mobile user. The problem with this strategy is the significant effort it takes to produce the grid. This method uses only the existing infrastructure at a site (eg, Wi-Fi access points).

EDIPS uses a combination of inertial navigation, triangulation, and fingerprinting. During the offline phase, the signal strengths are calculated based on only the Wi-Fi access point (as reference points) and a signal propagation model [23]. Based on this information, a discretization of the physical space is automatically performed. A grid with fixed-sized cells is overlapped onto the blueprint of the physical area. Every cell has preloaded the expected signal strengths assigned by the signal propagation model. By comparing the current signal strengths captured by a mobile device with the signals preloaded in the system, it is possible to determine the user location with minimal effort. EDIPS has fast deployment with an acceptable degree of precision (between 2 and 6 m) that makes it suitable for finding people in most indoor scenarios (eg, hospitals). Based on this application, the knowledge exchange can then be done through face-to-face interactions.

### The Integration of Tools

#### Conceptual Integration

Both MEK and EDIPS have different functions, although when combined they can increase their potential. Both applications work in a loosely related way; that is, the collaboration between users is on-demand and involves a short time period [25]. For instance, the devices of 2 users would remain connected only during the time required to perform the matching of user interests and the knowledge exchange.

For the integration of these tools, a mechanism for information exchange between these systems was proposed. It enriches the information from each application and supports collaboration. MEK provides information on the exchange of knowledge, while EDIPS contributes with the positioning of the participants involved in the exchange at the time it is performed.

**Figure 6:**
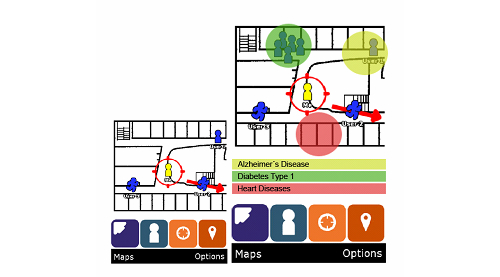
Screenshots of original EDIPS (left) and EDIPS incorporating information provided by MEK (right) [25].

Further analyses can be done by both applications with the information obtained from both of them. MEK may perform analyses involving the positioning of the participants, and it can highlight the location and amount of knowledge that has been exchanged at a certain location, for example. EDIPS can also interpret the data received from MEK and enrich it with points on the map representing knowledge areas (see Figure 6). It can be used to identify groups that have the same interests, the usual locations of these groups, and how people move in a certain physical area (eg, in a hospital). A breakdown of locations with their exchanged knowledge can be shown on EDIPS maps. This kind of visualization is appropriate to show from which users and in which areas one is more likely to find a piece of knowledge for a certain category.

This MEK-EDIPS integration can also be used in several contexts, especially in the health care scenario. The next section describes the use of this knowledge-sharing environment for various purposes in hospitals and clinics.

#### Example Scenarios

Health care is one possible scenario where the integrated MEK-EDIPS tool can be used. This scenario involves people who play several roles: physicians and other health professionals, patients (adults or children), patients’ families and friends, recovered patients, and managers of health centers. All these people are considered and supported by the proposed environment. The following example illustrates the support that can be provided by MEK-EDIPS to participants in a health care scenario.

Mary is undergoing breast cancer treatment. Today she is being consulted by her physician, Dr Silva, who identifies new symptoms and some unrecognized reactions. While Mary is waiting for a chemotherapy session, she uses MEK-EDIPS to receive information about her disease and reports from other women who also undergoing treatment for breast cancer. She takes the opportunity to ask a question about metastasis.

The chemotherapy session starts, her mobile phone is off. Her husband takes this time to learn about the usual fears of patients with breast cancer in his own phone. He asks some questions about it and through the integrated MEK-EDIPS tool he identifies Helen, a breast cancer survivor, who is under monitoring. Helen is available to contact and his device indicates that she is across the corridor, close to him. He walks across the hall, presents himself, and asks his questions. During this face-to-face conversation with Helen, his phone is receiving and sending pieces of knowledge about his main interests, “psychological effects of cancer” and “symptoms of depression.”

Simultaneously, a nurse elsewhere in the hospital identifies erroneous information about an alternative HIV treatment. She reports it and then enters the correct information. She also answers Mary’s question about metastasis.

During lunch break, Dr Silva identifies a group of experts at a nearby table through the integrated MEK-EDIPS tool. He goes to their table to clarify some concerns about Mary’s new symptoms and reactions. In the same restaurant, Dr Koothrappali, who has just come from an important conference, is telling other doctors via his phone some news about treatment options.

By using all the information exchanges supported by the integrated MEK-EDIPS tool, Dr Wilson, the hospital manager and head of the Oncology Department, recognizes that there are many questions about breast cancer. People who interact about this issue (asking questions or providing pieces of knowledge) are usually in the hospital on Mondays from 2 pm to 3 pm. Therefore, he organizes some lectures every Monday at 2:30 pm and distributes leaflets and notices to the health staff to identify the main questions from their patients.

#### Software Integration

**Figure 7:**
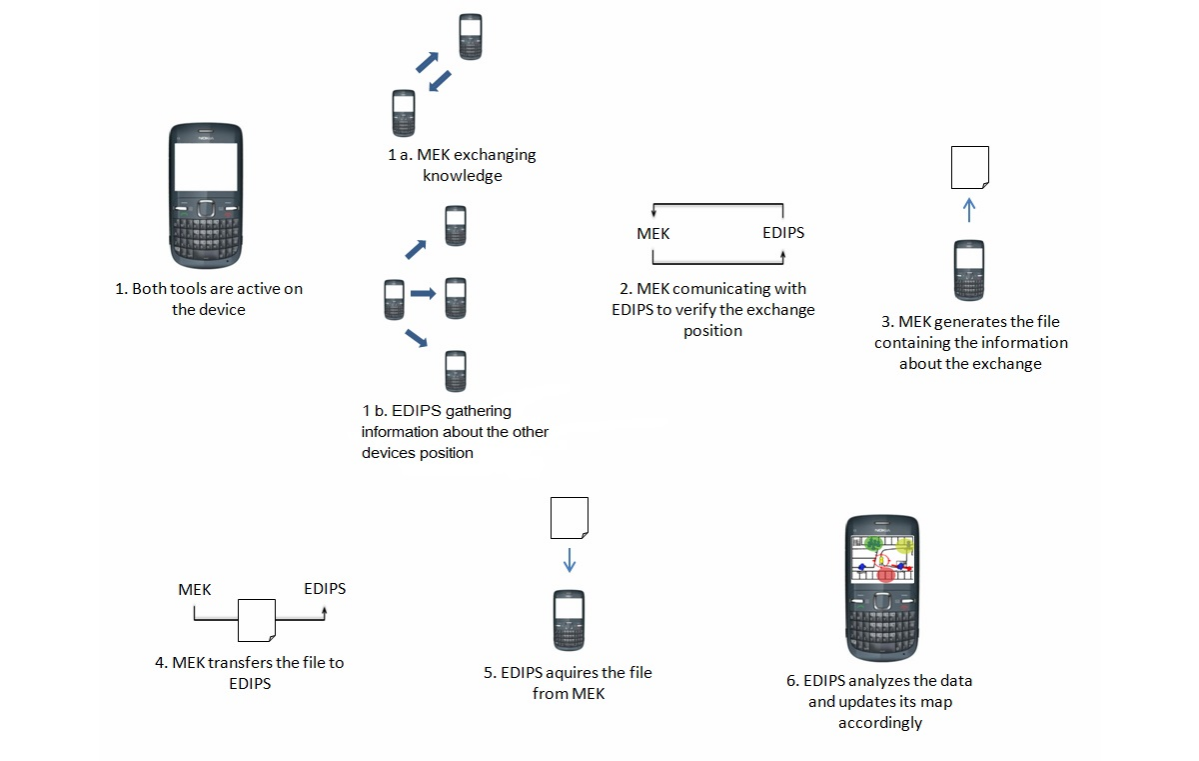
Integration of the apps.

The data integration between the 2 app (MEK and EDIPS) was possible through the exchange of extensible markup language (XML) files with the contextual information captured by each. This data format was chosen because it is lightweight, structured, and standardized, thus facilitating data interoperability. The proposed integration between MEK and EDIPS is depicted in Figure 7.

Both apps must be running simultaneously on the user’s mobile device. While MEK uses contextual information to perform knowledge exchange, EDIPS maps the position of other users. MEK searches for knowledge that may be useful to the user. When a piece of knowledge is found, the exchange is performed; that is, a copy of the found knowledge is transferred to the user’s mobile device. To perform this exchange, MEK needs to know the knowledge categories (from its taxonomy) that the user is interested in and some personal information from the user profile.

**Figure 8:**
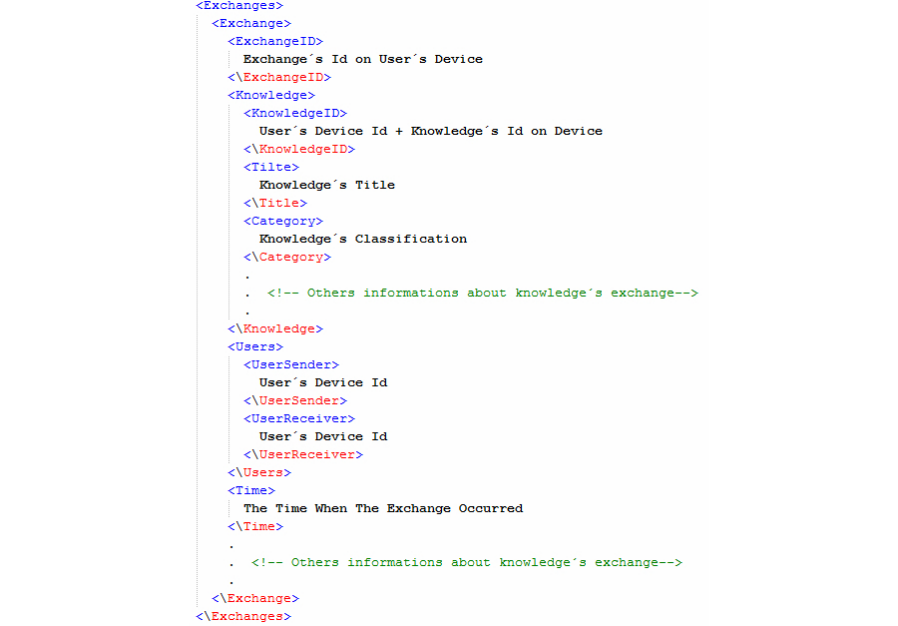
XML structure of the context file.

When the exchange occurs, MEK performs 2 tasks. The first task is to communicate with EDIPS to request the position of the device sending the knowledge. Once this information arrives, MEK performs the second task, which is the construction of the XML file with the information about the occurrence/location of the exchange. This metadata and information about the time when the transfer occurred and the participants in the exchange are stored in the XML file. A part of the XML file is shown in Figure 8.

The XML file can also be used as an input for EDIPS, providing additional information for updating its map and for MEK for the identification of groups of interest and statistics about knowledge exchange. Once the exchange occurs, MEK can run analyses about which location has the highest exchange rates. With this information, one can find out which areas have more users with similar interests.

The file generated in the previous step is relayed to EDIPS, which then takes the necessary data to improve its mapping. EDIPS also runs analyses of the data to add awareness information to the map. Boundaries for areas of interest may also be produced; that is, EDIPS can mark with different colors on its map the locations that concentrate a large number of people with a particular interest. Figure 6 shows 3 groups: light green, dark green, and red, which represent people interested in Alzheimer disease, type 1 diabetes, and heart disease, respectively. All this information will be displayed on the EDIPS screen, providing data to the end users in a visual and easy-to-understand manner.

## Results

### Analysis of Previous Work

#### Overview

The apps and articles selected to be part of the study corpus were grouped into 4 categories: social networking, mobile applications, online tools, and academic literature. We found social networking apps that would only be useful for patients and other apps that would only be useful for health professionals, but the main goal of both clusters was the exchange of experiences among users.

Some apps for patients focused on searching for people who have the same diseases, with the aim of sharing experiences among them. Furthermore, there were apps for treatments and activities that could help find information for patients and their families. Some apps for health professionals were used to exchange experiences related to patient’s medications and the discovery of new treatments.

#### Social Networking Tools

In the social networking area, the following tools were selected: Everyday Health [26], PatientsLikeMe [27], HealthVault [28], and Sermo [29].

Everyday Health [26] is a social network geared toward patients. The user can search for other people with the same interests in the health area and exchange experiences with them. The user states his/her interests when registering by choosing from a preset list of interests.

PatientsLikeMe [27] is a social network catering to patients. Users can search for other users with the same interests. Once found, one can follow other users to see what they have posted. There is a forum area to support interactions among users. It is mainly used to clarify questions and share experiences.

HealthVault [28] is a Microsoft tool that is based on a social network designed to facilitate learning in the health care area. The tool allows users to record personal information and medical records that can be accessed by authorized people or health centers in the event of emergencies.

Sermo [29] is a social network for health care professionals. It is for finding new procedures and exchanging experiences and results of disease treatments. To register with it, a user needs a credential that proves expertise in health care professional services. For this reason, the application was not formally considered in the evaluation process.

#### Mobile Apps

For mobile apps, we chose AsthmaMD [30] and Epocrates [31]. These apps take advantage of the diffusion capabilities of mobile devices to connect health professionals with patients (eg, to find a second opinion on a patient’s disease). In the case of Epocrates, it can also be used to help people find information about medications, dosages, and their collateral effects.

AsthmaMD [30] is a free application for mobile devices that allows users to register their asthmatic activities, medications, and the causes of their crises, much like a diary. This information can be shared with other users. Using this information, it is also possible for users to generate a graph with activities that can be shared with their doctors and stored in their health records. The application also promotes the openness of these files so that researchers can study the cases for scientific purposes. For this reason, the files are all anonymous.

Epocrates [31] is a large online database for mobile devices. This tool includes data on medication dosage, indications for the use of drugs, laboratory tests, adverse reactions, pharmaceutical information, and other clinical data and papers on the topic. As Epocrates focuses on drug information (and not in collaboration or information sharing among professionals), we did not include it in the comparison.

#### Online Tools

The third area of related works involved online tools that help patients search for a second medical opinion or advice from a health specialist. In this area, we selected an online tool named Doctle [32]. After registration, the user can initiate a new medical consultation to obtain an opinion or advice. This service is not free. Doctle [32] is an online tool that allows patients to get a medical consultation via the Internet. Although registration is free, the user has to pay to set an appointment or send his/her medical history to the physician.

#### Academic Literature

The academic literature category reported on initiatives for helping improve people’s well-being. These research works introduced tools or frameworks with a scientific reasoning [33-37].

HOMEVMI [33] is a tool that can give advice to its users and deliver alerts regarding their lifestyle. The advice is personalized according to the user’s input and other conditions identified by sensors connected to the patient.

Wei and Yang [34] designed an app that allows patients to access the server of a hospital to retrieve information or suggestions on diseases and symptoms. Physicians can also monitor their patients using the system.

Benavides et al [35] described a mobile application that uses Bluetooth to discover and record users who are interested in similar health topics. This can be used to infer the proximity of those people, to detect contact between 2 people, the duration of the contact, and he geolocational data. The contact data are then used to evaluate the potential spread of an infectious disease—a transmission vector represents the proximity to infected agents.

Ramos et al [36] reported on an app for mobile devices that enhances communication between doctors and patients who live in distant areas with difficult access.

MobiClique [37] is middleware for mobile social networking. This tool allows for studying the behavior of mobile social networks and interactions among people, based on opportunistic communication by using Bluetooth.

### Comparison of Functionality

We compared the functions provided by these systems and the objectives of the software. The findings of the comparison process described previously as well as the integrated MEK-EDIPS tool are presented in Tables 1 and 2.

**Table 1:** Current social networking tools, mobile apps, and online programs: Analysis from Medicine 2.0 perspectives.

Requirements		Work^a^			
		Everyday Health	PatientsLikeMe	HealthVault	Doctle
**Social networking**					
	Relationships and interaction types	–	–	–	–
	Network structure	+	+	–	–
	Egocentric network	+	+	–	–
	Graph metrics	N/A	N/A	N/A	–
	Relevant members	+	+	–	–
	Content and main topics	+	N/A	–	–
**Participation**					
	Asynchronous communication	+	+	+	+
	Synchronous communication	–	–	–	–
	Mechanisms for encouraging participation	–	+	–	–
	Promotion of various kinds of participation	–	–	–	–
	Security and privacy of the user’s personal information	+	+	–	+
	Information protection	+	–	N/A	–
	Interest identification	+	+	–	–
	Expertise identification	–	–	–	–
	Attention level	–	–	N/A	–
**Apomediation**					
	Autonomous operation	+	+	–	–
	Decentralized environment	+	+	–	–
	Informal learning	+	+	+	N/A
	Synchronous communication	–	–	N/A	–
	Message credibility	N/A	N/A	N/A	–
	Information filtering	N/A	N/A	N/A	–
	Detection of opinion leaders	–	–	N/A	–
**Collaboration**					
	Location awareness	–	–	–	–
	Contextual information	+	+	N/A	–
	Opportunistic collaboration	+	+	–	–
**Openness**					
	Content formats	–	N/A	N/A	–
	Semantic integration	N/A	N/A	N/A	–
	Transparency	+	+	–	–
	Free access	+	+	+	–

^a^+: the application incorporates the attribute; –: the attribute is not incorporated; N/A: the attribute analyzed was not found or it was not mentioned in the paper or website of the tool.

**Table 2:** Proposed works: Analysis from Medicine 2.0 perspectives.

Requirements		Work^a^			
		HOMEVMI	Ramos et al [36]	Benavides et al [35]	MEK-EDIPS
**Social networking**					
	Relationships and interaction types	+	–	–	+
	Network structure	–	–	+	–
	Egocentric network	–	N/A	+	+
	Graph metrics	–	–	N/A	–
	Relevant members	+	–	N/A	+
	Content and main topics	–	–	–	+
**Participation**					
	Asynchronous communication	+	+	–	+
	Synchronous communication	–	+	–	+
	Mechanisms for encouraging participation	+	–	–	+
	Promotion of various kinds of participation	+	–	–	+
	Security and privacy of the user’s personal information	N/A	–	–	+
	Information protection	N/A	N/A	–	+
	Interest identification	N/A	–	–	+
	Expertise identification	+	–	–	+
	Attention level	–	–	–	+
**Apomediation**					
	Autonomous operation	+	+	–	+
	Decentralized environment	+	–	–	+
	Informal learning	+	–	–	+
	Synchronous communication	N/A	–	–	+
	Message credibility	N/A	–	–	+
	Information filtering	–	–	–	+
	Detection of opinion leaders	–	–	–	+
**Collaboration**					
	Location awareness	N/A	N/A	–	+
	Contextual information	+	+	+	+
	Opportunistic collaboration	–	–	–	+
**Openness**					
	Content formats	N/A	+	–	+
	Semantic integration	–	–	–	+
	Transparency	+	+	–	+
	Free access	N/A	N/A	+	+

^a^ +: the application incorporates the attribute; –: the attribute is not incorporated; N/A: the attribute analyzed was not found or it was not mentioned in the paper or website of the tool.

We can see in Tables 1 and 2 that the presented works do not support the social networking and the participation. The MEK-EDIPS provides a good support to these requirements, as to the others.

For the integrated MEK-EDIPS tool, the focus is on knowledge dissemination. The users’ relationships and interactions types can be information exchange, chatting, or face-to-face meetings. The user can identify the frequency of his/her interactions, who participates in them, when they occur, and the type of communication (egocentric network). Network structure and Graph metrics are requirements missed in MEK-EDIPS, because this solution does not provide mechanisms to analyze the complete social network. It was not part of its design goals, but the social information can be easily exported to a social network analysis tool, as example Gephi.. The most relevant members can be identified by the frequency of participations; therefore, relevance is related to the degree of cooperation. The categories of a taxonomy and keywords used by users to describe the resources and their own interests are related to identification of content and main topics interactions.

The integrated MEK-EDIPS tool provides both asynchronous and synchronous communications. This solution does not have very sophisticated mechanisms for encouraging participation—it is achieved in a proactive way because people have learning needs and gaps in their knowledge. Receiving useful information provides interesting benefits, such as clarifying and filling in the knowledge gaps, or it can be used to find and interact with people who have the same interests or are experts in these areas.

The integrated MEK-EDIPS tool promotes some kinds of participation, such as information sharing, validation, chatting, and face-to-face meetings. Because the main goal of the integrated MEK-EDIPS tool is the dissemination of information, none of the user’s personal information is available (security and privacy), and his/her resources can be public or private (information protection). As explained previously, this solution allows the identification of the users’ areas of preference (interest identification) and the identification of experts (expertise identification). In connection with MEK, information sharing is transparent to users, whereas in EDIPS, the user can be invisible if one does not wish to be interrupted or identified (attention level).

The conceptual base of MEK is apomediation. The users determine information creation and provision (autonomous operation) in a decentralized environment, using their mobile devices. The learning process is fully participative and collaborative. It involves the use of official and unofficial sources (informal learning), and the crowd evaluates them in a continuous process (synchronous communication). All the content is provided for and by common people, mostly nonexperts, based on their own understanding or experiences (message credibility). During this process of information creation and dissemination, all the members of this huge network filter useful information (information filtering), validating, and commenting on each resource. Opinion leaders can be detected by the frequency of their collaboration and participation.

Indoor location awareness is the main focus of EDIPS. It allows identification of people in a physical space, and following and interacting with people who are in close proximity. The integration with MEK enables the identification of people with similar interests. The contextual information used to enrich content and empower collaboration is as follows: time (when the knowledge was created, an interaction occurred, or people passed by a specific location), spatial information (usually maps), areas of interest, and information content. In its creation, MEK was based on opportunistic collaboration.

The integrated MEK-EDIPS tool uses any data that can be scanned or digitally created; for example, images, text, or audio (content formats). The information type can be identified using a common taxonomy (semantic integration). If this information is available in the knowledge sharing application, it is considered to be accessible data (transparency) that can be shared for free (free access).

After analyzing the proposals that are part of the study corpus, it is possible to say that there is no work supporting a complete knowledge flow among the participants in the health care process, according to the guidelines given by Medicine 2.0 principles. This evidence leads us to expect an interesting contribution from the integrated MEK-EDIPS tool as a supporting tool for the entire knowledge flow process (as shown in Figure 1).

### Reliability Evaluation

#### MEK and EDIPS

We evaluated the MEK capability to support knowledge sharing among participants in the health care process. We tested the capability of EDIPS to locate people in order to promote face-to-face interactions among them, based on their common interests.

#### MEK Evaluation

**Figure 9:**
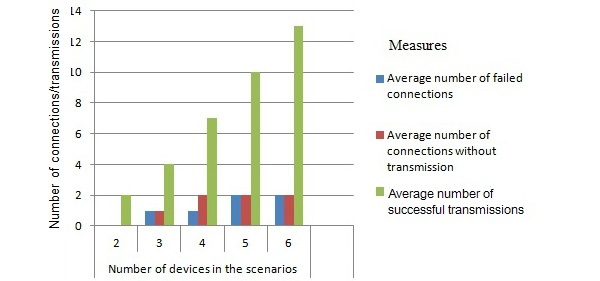
Results of reliability evaluation of MEK.

Figure 9 shows the results obtained in the reliability evaluation of MEK. The x-axis represents the number of devices used in each scenario described previously, whereas the y-axis shows the average number for each measurement.

In these tests, the precision rate was 100% and recall rate was 80%. As shown in Figure 9, the number of failed connections or connections without transmission was very low compared to the successful knowledge exchange interactions.

#### EDIPS Evaluation

The results obtained in the EDIPS positioning evaluation are presented in Table 3. The average distance error across all 7 testing locations was <6.28 m, an acceptable distance if we are trying to find people based on such information.

**Table 3:** Average distance errors and standard deviations for the EDIPS testing locations.

Location	Error distance (m), mean (SD)
1	4.57 (2.02)
2	3.63 (2.07)
3	6.25 (1.12)
4	5.74 (2.31)
5	6.28 (2.15)
6	6.16 (2.63)
7	4.27 (1.83)

### Usability and Usefulness Evaluation

#### Sample Characteristics

To understand how professionals usually acquire knowledge, what they consider important information during a treatment, and how their patients learn about their diseases and cures, we gave a questionnaire to a sample of health professionals. In this study, 18 health professionals participated: 14 females and 4 males. We present their ages, occupations, the place where they work (some researchers work in hospitals), and their experience (in years) in Table 4.

**Table 4:** Participants’ characteristics (N=18).

Characteristics		n (%)
**Gender**		
	Male	4 (22)
	Female	14 (78)
**Age (years)**		
	≤30	8 (45)
	31-40	3 (17)
	41-50	5 (28)
	>51	2 (11)
**Occupation**		
	Physician	7 (39)
	Medical student (in final year)	1 (6)
	Physiotherapist	1 (6)
	Nurse	5 (28)
	Psychologist	1 (6)
	Dentist	2 (11)
	Nursing assistant	1 (6)
**Workplace**		
	Clinic	10 (56)
	Research institute or university	2 (11)
	Other	6 (33)
**Experience (years)**		
	<5	6 (33)
	5-10	5 (28)
	11-15	2 (11)
	16-20	1 (6)
	21-25	1 (6)
	>25	3 (17)

Although there is an imbalance in the age and gender of the participants because participants are not distributed equally using these variables. It is not a problem because these variables are not particularly important for this experience. We were looking for people with some relevant professional expertise in different health care areas. For this study, the variables related to expertise (ie, experience, academic background, and occupation) are more relevant. Concerning the occupations, we preferred professionals who interacted directly with patients, performed clinical work, and other kinds of assistance.

In all, 13 participants were specialists in some of the following areas: mental health and psychosocial care/emergency, trauma orthopedics, pediatrics, anesthesiology, family and community medicine, nephrology, organ donation and transplants, or pathology. Moreover, 56% (10/18) of our sample worked directly with the treatment of diseases (clinic), 11% (2/18) in research, and 33% (6/18) had other kinds of assignments, such as intensive care units or emergency and pathology diagnosis.

Regarding the participants’ experience (Table 4), 39% (7/18) had more than 10 years of experience, 28% (5/18) had between 5 and 10 years, and 33% (5/18) had less than 5 years of experience. Concerning their academic background in addition to being an MD, 33% (6/18) held a PhD degree, 11% (2/18) held a Master’s degree, 33% (6/18) held a specialized training or  residency, 17% (3/18) were graduate students, and 5% (1/18) had a technical degree. Taking into account these characteristics, we considered it to be a valid sample.

#### Domain Comprehension: How Professionals and Patients Learn

Using this sample, we understood how the participants (and their patients) create knowledge and identified some characteristics of this process (eg, frequency, main information sources, and source reliability). The participants answered the questions presented in the questionnaire and also provided extra information about this process. The main information sources with their most common usage frequency are:

1. Classroom/face-to-face courses: Most participants use this information source, but with low frequency (at least once a year).
2. Lectures: Similar to the previous one, many people sporadically use this knowledge source.
3. Discussion with professionals outside my area and specialized websites: Both sources are used most commonly from 1 to 3 times per month.
4. Scientific articles and discussion with less experienced professionals: These sources are frequently used (most commonly from 1 to 3 times per week).
5. Textbooks and empirical observation of other professionals: These are the most frequently used knowledge sources. Both are most commonly used daily or almost daily.

Table 5 presents the main information sources used by health professionals to create, validate, or update knowledge, and the frequency with which they used those sources.

**Table 5:** Sources of participants’ knowledge acquisition (N=18).

Resource used in knowledge acquisition	Usage frequency, n (%)				
	>1 time/year	2-6 times/year	1-3 times/month	1-3 times/week	Daily
Classroom/face-to-face courses	13 (72)	2 (11)	0 (0)	2 (11)	0 (0)
Distance courses	5 (28)	2 (11)	1 (6)	0 (0)	0 (0)
Lectures	1 (6)	11 (61)	3 (17)	2 (11)	0 (0)
Textbooks	0 (0)	3 (17)	5 (28)	4 (22)	5 (28)
Scientific articles	0 (0)	3 (17)	5 (28)	6 (33)	3 (17)
Discussion with more experienced professionals	0 (0)	3 (17)	5 (28)	5 (28)	4 (22)
Discussion with less experienced professionals	1 (6)	3 (17)	2 (11)	6 (33)	4 (22)
Discussion with professionals outside of my area	1 (6)	4 (22)	6 (33)	2 (11)	4 (22)
Presentations in scientific meetings	4 (22)	7 (39)	1 (6)	0 (0)	1 (6)
Empirical observation of other professionals	2 (11)	5 (28)	1 (6)	4 (22)	5 (28)
Specialized websites	0 (0)	3 (17)	6 (33)	4 (22)	4 (22)
Study groups (face-to-face or virtual)	2 (11)	5 (28)	4 (22)	1 (6)	1 (6)
Social networks or media	4 (22)	1 (6)	4 (22)	0 (0)	3 (17)
Other	0 (0)	2 (11)	0 (0)	0 (0)	0 (0)

During a treatment, it is important that the health professional can get information from the patient to understand their health situation. Table 6 shows the importance level of information about a certain element of a patient’s disease or treatment according to the opinions of the participants.

Symptoms were rated by the participants as extremely important information, the treatments already undertaken were considered to be very important, and religious beliefs and superstitions and details about work were regarded as important issues. Moreover, the participants mentioned other kinds of relevant information, such as the quality of family relationships or the social behavior of the patient.

The participants were also asked about how easy it is to get important information from the patient. The results obtained indicate that it is not a difficult task (Table 6). The main difficulties were related to access to personal information; for example, details about work or residence, or information about the private life of the patients.

**Table 6:** Ratings of importance of information for a treatment and difficulty gathering information from patients (N=18).

Relevant topics for a treatment	Importance, n (%)^a^					Difficulty, n (%)				
	1	2	3	4	5	1	2	3	4	5
Symptoms	0 (0)	0 (0)	0 (0)	0 (0)	16 (89)	1 (6)	2 (11)	5 (28)	5 (28)	3 (17)
Doubts about the disease	0 (0)	0 (0)	2 (11)	3 (17)	11 (61)	0 (0)	2 (11)	9 (50)	3 (17)	2 (11)
Fears about medication or treatment stages	1 (6)	0 (0)	2 (11)	5 (28)	8 (44)	1 (6)	3 (17)	8 (44)	2 (11)	2 (11)
Physical reactions	1 (6)	0 (0)	1 (6)	4 (22)	10 (56)	0 (0)	3 (17)	6 (33)	3 (17)	3 (17)
Psychological reactions	0 (0)	1 (6)	1 (6)	4 (22)	10 (56)	0 (0)	3 (17)	9 (50)	2 (11)	1 (6)
Previous diseases	0 (0)	0 (0)	2 (11)	5 (28)	9 (50)	0 (0)	3 (17)	4 (22)	5 (28)	3 (17)
Treatments already undertaken	0 (0)	0 (0)	3 (17)	7 (39)	6 (33)	0 (0)	4 (22)	5 (28)	2 (11)	4 (22)
Routines, hobbies, and information on private life	0 (0)	1 (6)	6 (33)	3 (17)	6 (33)	1 (6)	5 (28)	7 (39)	0 (0)	2 (11)
Religious beliefs and superstitions	1 (6)	1 (6)	9 (50)	3 (17)	2 (11)	1 (6)	2 (11)	8 (44)	2 (11)	1 (6)
Details about work (eg, location, infrastructure, and level of violence)	0 (0)	0 (0)	9 (50)	2 (11)	5 (28)	0 (0)	6 (33)	7 (39)	2 (11)	0 (0)
Details about residence (eg, location, infrastructure, basic sanitation, transportation, and level of violence)	0 (0)	0 (0)	8 (44)	3 (17)	5 (28)	1 (6)	6 (33)	7 (39)	1 (6)	0 (0)
Educational and cultural background	1 (6)	2 (11)	7 (39)	1 (6)	5 (28)	0 (0)	2 (11)	8 (44)	2 (11)	3 (17)
Other	0 (0)	0 (0)	0 (0)	0 (0)	4 (22)	0 (0)	2 (11)	2 (11)	0 (0)	0 (0)

^a^1=Not important, 2=somewhat important, 3=important, 4=very important, 5=extremely important.

^b^1=Difficult, 2=somewhat easy, 3=easy, 4=very easy, 5=extremely easy.

According to the health care professionals’ opinions, the most reliable information sources for the patients are were their friends and relatives, or people who have the same disease (both recorded very frequent use). Although they are the most frequent mechanisms to solve patients’ doubts, they are not considered reliable sources (Table 7). When asked for other sources, the sample mentioned newspapers and magazines, which are also not reliable sources.

The participants were asked if they believe that greater interaction among patients, professionals (eg, doctors, nurses, aides), researchers, recovered patients, and caregivers (eg, family and friends) could benefit the acquisition, validation, and exchange of knowledge on the patient’s disease or treatment. Of the total participants, 94% (17/18) answered yes and 6% (1/18) said no.

**Table 7:** Reliability level of information sources according to health care professionals.

Information source	Reliability level, n (%)^a^					Frequency of access to the information source, n (%)^b^				
	1	2	3	4	5	1	2	3	4	5
Scientific publication	0 (0)	0 (0)	2 (11)	7 (39)	4 (22)	4 (22)	2 (11)	0 (0)	1 (6)	1 (6)
Other health professionals	0 (0)	0 (0)	9 (50)	2 (11)	3 (17)	0 (0)	6 (33)	4 (22)	1 (6)	3 (17)
Friends and relatives	3 (17)	4 (22)	6 (33)	0 (0)	0 (0)	0 (0)	1 (6)	3 (17)	4 (22)	6 (33)
Known people who have had the disease	2 (11)	3 (17)	7 (39)	2 (11)	0 (0)	1 (6)	1 (6)	1 (6)	6 (33)	5 (28)
Friends and relatives of people who have had the disease	3 (17)	5 (28)	6 (33)	0 (0)	0 (0)	1 (6)	1 (6)	2 (11)	4 (22)	6 (33)
Social networks or media	5 (28)	6 (33)	2 (11)	0 (0)	0 (0)	1 (6)	6 (33)	2 (11)	2 (11)	3 (17)
Webpages and other Internet materials	1 (6)	7 (39)	5 (28)	0 (0)	0 (0)	1 (6)	3 (17)	3 (17)	4 (22)	2 (11)
Specialized virtual communities (focused on the disease)	2 (11)	1 (6)	8 (44)	3 (17)	0 (0)	4 (22)	3 (17)	4 (22)	2 (11)	1 (6)
Other	0 (0)	2 (11)	0 (0)	0 (0)	0 (0)	0 (0)	1 (6)	0 (0)	0 (0)	0 (0)

^a^ 1=unreliable, 2=not very reliable, 3=reliable, 4=very reliable, 5=extremely reliable.

^b^ 1=very little use, 2=little use, 3=regular use, 4=frequent use, 5=very frequent use.

#### Proof of Concept

The integrated MEK-EDIPS tool was presented to the participants. Then, they determined how important it would be to use integrated MEK-EDIPS tool to support certain activities of the members of a health care community (Table 8).

The use of the integrated MEK-EDIPS tool was considered extremely important to support the activities presented in Table 8. The participants mentioned that the integrated MEK-EDIPS tool facilitates the search for professional content. Moreover, 67% (12/18) would like to use or would recommend the use of this environment. Therefore, this supports our hypothesis that there are recognized possibilities for using the integrated MEK-EDIPS tool and the use is considered important and would be recommended.

**Table 8:** Importance of the integrated MEK-EDIPS tool in the health scenario.

Activities to be supported		Level of importance, n (%)				
		A little important	Somewhat important	Important	Very important	Extremely important
**For patients and relatives**						
	Obtaining information on the disease, additional understanding, and, consequently, better treatment	0 (0)	1 (6)	3 (17)	4 (22)	7 (39)
	Facilitating the interaction with people who are going through, or have gone through the same illness	0 (0)	0 (0)	4 (22)	4 (22)	7 (39)
	Improving the reliability of the information that they get. This is an expected result because the shared information is read and evaluated by a larger number of people (some of them could be specialists)	0 (0)	3 (17)	2 (11)	3 (17)	7 (39)
**For health professionals**						
	Possibility to more easily expand their knowledge by obtaining additional scientific information, articles, results of experiments, and procedures provided by specialists in an area of interest	0 (0)	3 (17)	1 (6)	2 (11)	9 (50)
	Obtaining information that may help in the treatment, but that is usually omitted in consultations; for example, major doubts, unreliable data the patient may rely upon, reactions, beliefs, etc	0 (0)	1 (6)	3 (17)	2 (11)	9 (50)
**For researchers**						
	Collecting information or results to help them create new hypotheses and do further research	0 (0)	1 (6)	4 (22)	2 (11)	8 (44)
**For managers**						
	Improving the provision of health information based on the identification of the most interesting topics for patients and health professionals	0 (0)	1 (6)	4 (22)	2 (11)	8 (44)

The participants were asked if they believe that this environment can increase the knowledge flow in health environments. They used a 5-point scale to indicate their answer, from 1=never” to 5=always.” The results indicate that 22% (4/18), 11% (2/18), and 39% (7/18) of participants think it can increase knowledge flow sometimes, often, and always, respectively. This supports our hypothesis that the integrated MEK-EDIPS tool can increase the knowledge flow in a health scenario.

The participants indicated that the proposed environment has strengths and limitations. The most important strengths were:

1. Improvement of doctor-patient interaction.
2. Optimization of time in communication with patients. Consequently, this time can be used to better evaluate the patients and choose the best treatments.
3. Clarification of professional doubts quickly, thereby reducing the risks of forgetting to look for an answer.
4. Support for new doctors who may be reluctant to ask someone with more experience.
5. Helping healthy people get data about health, and also act in disease prevention.
6. Knowledge dissemination to be made easier, and allows the exchange of ideas and diffusion of scientific knowledge.
7. Improvement of the use in home care services.
8. Acquiring of knowledge in a more accurate and efficient way, because other professionals check the accuracy of the shared information.
9. Exchanging of relevant information, especially on rare and serious diseases.

The participants also mentioned several limitations of the proposed environment . These included a possible lack of privacy, inexperience in the use of the system could be a problem to lay users, there is no guarantee that the information sources are reliable, there are technical limitations if the users utilize a smartphone without Android or Windows Phone, and the hardware requirements for using the system could restrict its usage for socioeconomically disadvantaged people.

## Discussion

### Principal Findings

In this work, we analyzed how the flow of knowledge occurs in a health care scenario and the benefits that it brings to the people involved. Considering the Medicine 2.0 paradigm, we analyzed the scientific proposals and software apps that could contribute to support this new health care paradigm.

The review of previous work unveiled the current landscape and their main proposals and limitations. This review process also allowed us to build a list of requirements that can be used to guide the design and development of future solutions. Because none of the analyzed solutions supported all of the main concepts involved in Medicine 2.0, we decided to develop a software environment that did support them.

Using a requirement list, the authors’ previous works, and taking advantage of the mobile computing paradigm, we developed a knowledge-sharing environment based on the integration of MEK [21,22] and EDIPS [23]. The suitability of this platform to support social networking, participation, apomediation, collaboration, and openness was evaluated with a sample of health professionals. The results obtained indicate that the proposed tool can be used to support most activities considered by health professionals as important in Medicine 2.0.

A precision rate of 100% and a recall rate of 80% were obtained in the proof of concept performed with the health professionals. Simulated situations for spontaneous collaboration among some of the participants were defined. In all cases, the participants were able to identify the physical location of their collaborator using the positioning service provided by the system. The maximum average error of this positioning service was 6.28 m.

According to the participants’, the proposed environment is both usable and useful. Of the total participants, 94% (17/18) agreed that the tool facilitates the knowledge flow among members of a health community (eg, patients, caregivers, doctors, and patients’ relatives). Most of them rated the use of the tool as extremely important in such a scenario. Of the total participants, 72% (13/18) thought that the system helps increase the knowledge flow, and 67% (12/18) would like to or would recommend its use. After comparing MEK-EDIPS to the other related apps and proposals, we believe that the integrated MEK-EDIPS tool is a good solution to facilitate the knowledge flow.

Concerning the support for knowledge sharing, the wireless communication mechanism used by the proposed integrated MEK-EDIPS tool is safe. The knowledge is only exchanged among devices running the system. In other words, there is no risk of a piece of knowledge being exchanged indiscriminately with other devices because the devices were accidentally paired by Bluetooth. However, this is an opportunistic collaborative approach, which, based on similar profiles, is characteristic of virally spread information. In this case, we cannot guarantee information reliability, inappropriate, abusive information, or erroneous content may be disseminated on the P2P network. At the same time, this problem can be attenuated by report functionalities and the removal of the content from the devices. Users can infer the accuracy of the information by analyzing the reliability of the information sources or feedback provided by other users.

In a knowledge-sharing network, we have members with different expertise and levels of education acting in different scenarios. Consequently, they have different languages and use specific vocabularies and terms. For example, biomedical researchers, doctors, and patients/lay people have different terms and understandings for the same concept. In our approach, all content can be classified as a concept of taxonomy and related to a set of tags. Moreover, we use different ways to recommend pieces of knowledge, envisioning a decrease in this problem. The different recommendation aspects are reported by Souza et al [21].

### Limitations

The integrated MEK-EDIPS tool has some limitations. For instance, it is focused on knowledge dissemination in a collaborative way among people involved in a particular treatment. In a health care scenario, there are solutions that incorporate other elements that were not considered in the proposed environment; for example, specialized components of the Internet of Things [38,39] and environmentally assisted living [40,41] paradigms. Solutions addressing computing paradigms usually involve specialized devices, which is exactly what we were trying to avoid.

If the tool is not available for all operating systems, the users have various restrictions regarding the type of device that they can use. The system uses Bluetooth to support the knowledge exchange; therefore, it can do it only if the involved devices are in communication range (iet, the distance between the information provider and the consumer is not more than 10 m).

In the systematic review, we excluded all apps that were not freeware because commercial products were out of the scope of this work. This does not exclude the possibility of finding commercial products similar to the proposed environment.

Concerning the experiments, although we consider our sample to be valid for the previously explained reasons, it is small and may not represent the whole population. Because we based our study on free participation (ie, we invited a large number of health professionals who could freely refuse or accept to participate in our experiment), it is typical to have low participation rate. Usually, a sample based on free participation is more responsible and dedicated. In this initial assessment, we preferred using a small but involved sample.

For the same reason, we did not involve patients in this initial assessment. Considering that we would only do a proof of concept study, the participation of health professionals, who have intensive contact with patients was deemed appropriate. This proposal does not address the social aspects involved in the health scenario or in the knowledge exchange process. For instance, it is common that people with high levels of depression have no desire to interact with other patients or learn new things. Consequently, these people would not take advantage of our solution. Moreover, the proposed solution assumes that the users know how to utilize smartphones. This could represent a problem for elderly people. Related aspects that should also be addressed in the future are information privacy and trustworthiness. The main limitation of this proposal is the lack of a formal evaluation of its impact in a real scenario. However, this is an activity that is currently being addressed.

### Conclusions and Future Work

The proof of concept performed with health professionals gave us an interesting insight into the potential impact and limitations of this proposal. The integrated MEK-EDIPS tool could be used to support the different actors that participate in a health care process and also to make the search and retrieval of scientific knowledge easier. The impact of the system, as a support for people in a Medicine 2.0 scenario, was considered good by the participants in the proof of concept. Participants also considered the proposal as useful and usable. Although the system has strengths and weaknesses, its limitations have low relevance compared to the advantages that can be provided. Therefore, we envision that the proposed integrated MEK-EDIPS tool is a good first step toward the development of solutions supporting Medicine 2.0.

As future work, we will formally evaluate the integrated MEK-EDIPS tool in a real-world scenario to more accurately understand its strengths and weaknesses. Moreover, we will include Wi-Fi support to MEK, as a way of increasing the distance in which a knowledge exchange can be made between 2 devices. Thus, we will extend the coverage area of this tool.

## Abbreviations

EDIPS: Easy to Deploy Indoor Positioning System
GQM: goal, question, metric
IEEE: Institute of Electrical and Electronics Engineers
MEK: mobile exchange of knowledge
XML: extensible markup language
